# Learning Environments and Evidence-Based Practices in Bioengineering and Biomedical Engineering

**DOI:** 10.1007/s43683-021-00062-z

**Published:** 2022-01-31

**Authors:** Kristen Billiar, Donald P. Gaver, Kenneth Barbee, Anita Singh, John D. DesJardins, Beth Pruitt, Joe Tranquillo, Glenn Gaudette, Beth Winkelstein, Lee Makowski, Jennifer R. Amos, Ann Saterbak, Joe LeDoux, Brian Helmke, Michele Grimm, Paul Benkeser, LeAnn Dourte Segan, Bryan Pfister, David Meaney, Treena Arinzeh, Susan Margulies

**Affiliations:** 1 Department of Biomedical Engineering, Worcester Polytechnic Institute, Worcester, MA, USA; 2 Department of Biomedical Engineering, Tulane University, New Orleans, LA, USA; 3 School of Biomedical Engineering, Science and Health Systems, Drexel University, Philadelphia, PA, USA; 4 Department of Biomedical Engineering, Widener University, Chester, PA, USA; 5 Department of Bioengineering, Clemson University, Clemson, SC, USA; 6 Mechanical Engineering, and BioMolecular Science and Engineering Programs, University of California, Santa Barbara, Santa Barbara, CA, USA; 7 Departments of Biomedical and Electrical Engineering, Bucknell University, Lewisburg, PA, USA; 8 Department of Bioengineering, University of Pennsylvania, Philadelphia, PA, USA; 9 Department of Bioengineering, Northeastern University, Boston, MA, USA; 10 Department of Bioengineering, University of Illinois Urbana-Champaign, Urbana, IL, USA; 11 Department of Biomedical Engineering, Duke University, Durham, NC, USA; 12 Wallace H. Coulter Department of Biomedical Engineering, Georgia Institute of Technology and Emory University, Atlanta, GA, USA; 13 Department of Biomedical Engineering, University of Virginia, Charlottesville, VA, USA; 14 Department of Mechanical Engineering, Michigan State University, East Lansing, MI, USA; 15 Department of Biomedical Engineering, New Jersey Institute of Technology, Newark, NJ, USA; 16 Department of Engineering, Boston College, Chestnut Hill, MA 02467, USA

**Keywords:** Experiential learning, Team-based learning, Project-based learning, Problem-basedlearning, Learning environments

## Abstract

This paper provides a synopsis of discussions related to the Learning Environments track of the Fourth BME Education Summit held at Case Western Reserve University in Cleveland, Ohio in May 2019. This summit was organized by the Council of Chairs of Bioengineering and Biomedical Engineering, and participants included over 300 faculty members from 100+ accredited undergraduate programs. The Learning Environments track had six interactive workshops that provided facilitated discussion and provide recommendations in the areas of: (1) Authentic project/problem identification in clinical, industrial, and global settings, (2) Experiential problem/project-based learning within courses, (3) Experiential learning in co-curricular learning settings, (4) Team-based learning, (5) Teaching to reach a diverse classroom, and (6) innovative platforms and pedagogy. A summary of the findings, best practices and recommendations from each of the workshops is provided under separate headings below, and a list of resources is provided at the end of this paper.

## INTRODUCTION

Prior to the Education Summit, a number of surveys were conducted of Bioengineering and Biomedical Engineering (hereafter referred to as BME) department chairs. This organizational survey revealed that many topics of interest fell under the general theme of how to optimize the effectiveness and equity and access to educate *all* BME students, an issue that was described as Learning Environments, which is the subject of this paper. The responders identified the need to provide students with experiences to prepare them to identify and solve important global challenges in the teams of the future.

Several aspects emerged as especially prevalent and/or important training opportunities in BME. By its nature, BME is broad and interdisciplinary and attracts a broader demographic than other engineering disciplines. Work in the field therefore requires multi(sub)disciplinary teams in diverse (clinical, medical device, biotech, lab) environments to identify and tackle authentic problems using a structured engineering design process.

Two plenary lectures initiated the discussion of learning environments. In his plenary lecture, Robert Linsenmeier described the VaNTH (Vanderbilt-Northwestern-Texas-Harvard/MIT) Engineering Research Center program. This NSF-funded consortium program focused on ‘challenge-based education,’ which was used to teaching specific concepts in a way that motivated students by making them relevant to real world applications.^11^ He used the data from VaNTH and more recent surveys to demonstrate how BME curricula have changed over the past few decades, which showed commonality in approaches and the emergence of a core curriculum.^26,73^ In her plenary lecture, Wendy Newstetter described advanced problem-based learning (PBL) techniques that integrate the design of curricular and physical learning spaces to facilitate learning.^38^ PBL, originally developed to teach medical students in a more holistic way than through a series of theoretical courses, was adopted early in BME curricula. The adoption of PBL in BME was led in large part by Dr. Newstetter and her colleagues at Georgia Tech.^37^

To assist translation of innovative educational pedagogy to broad practice in many BME department, six interactive workshops engaged faculty to share best-practices in Learning Environments. Pre-meeting surveys were sent to participants of the workshops, and these data are provided in the general findings and discussion from the workshops that are described below.

## WORKSHOP 1. AUTHENTIC NEEDS IDENTIFICATION IN CLINICAL, INDUSTRIAL, AND GLOBAL SETTINGS

(Breakout Leaders: Drs. Kenneth Barbee, Anita Singh, and John DesJardins)

The goal of this breakout was to explore approaches for providing students real-world settings in which they could identify authentic problems to be solved in their course and/or senior design projects. The organizers surveyed BME programs on their current practices with regard to providing exposure to clinical, industrial, and global contexts for needs identification and project mentorship. In the workshop, participants rated the relative importance of each context and the relative effectiveness of various approaches to providing exposure. The discussion focused on sharing approaches that have been successful in overcoming barriers to authentic problem identification.

Participants placed high importance on providing clinical^20^ and industrial^8^ context for student design projects. Specifically, most attendees reported integrating these clinical and industry exposures in their course learning outcomes. Examples include:

- Applying engineering design to produce solutions that meet specific needs with consideration of global factors;
- Recognizing ethical and professional responsibilities in global contexts;
- Communicating effectively with a large audience, and
- Functioning effectively on a team.

However, despite the perceived effectiveness of immersive experiences for authentic needs-finding, pre-meeting survey results indicated that relatively few students (less than 25% of students in 82% of the reported schools) were getting access to these opportunities.

The discussion focused on effective alternatives to immersive experiences and novel approaches to overcoming barriers to implementation of programs. Expanded programs would enhance exposure to clinical and industrial settings for better preparedness of a future BME workforce. Each environment is discussed separately below.

### Clinical Exposure

There was general agreement that needs identification was best achieved through immersion in a clinical setting when compared to shadowing, lecturing or mentoring only. Barriers to providing clinical experiences for students cited by participants included lack of a collaborating medical center, difficulty with access to clinical settings (logistics, credentialing students, *etc*.), and the number of students in the program. Approaches to overcoming these challenges fell into three categories:

1. Expanding opportunities for immersion and needs-finding:
  A promising strategy, especially for schools without a large academic medical center, was to look at other types of facilities such as community health providers, rehabilitation centers, nursing homes and other assisted living environments. These alternative venues to the hospital setting offer expanded opportunities for needs finding related to health and wellness across the continuum of care.
2. Making the most of the available access:
  - Instances where only a limited number of students had an immersive experience can still serve as an indirect clinical immersion tool where the immersed students can lead teams of non-immersed students and offer insight from their clinical experience.
  - The needs-finding experience of a few students may generate many needs that can be shared with the students who did not participate in the experience.
  - If time in the clinic is limited, providing training on how to observe and interact with the various stakeholders can help to make most effective use of the time available.
3. Access to clinicians outside of the clinic:
  In lieu of direct access to the clinical settings, a forum for clinicians to discuss challenges they face and brainstorm with engineers on possible solutions can help with project identification and with matching student design teams with clinical mentors. Examples included:
  - Engineering Grand Rounds—students participate in discussion of cases.
  - Pitch Fairs—clinicians pitch project ideas for students to bid on.
  - Match.com-style pairing of clinicians to design teams.
  - Virtual access—watching videos of clinical procedures was suggested as an alternative to being present in person.

### Industrial Exposure

The participants agreed that co-op experiences provide better opportunities for authentic needs-finding in industrial settings when compared to lecturing and mentoring. Access to immersion experiences in industrial settings in the form of co-ops or internships varied widely among institutions, with over 40% reporting having no co-op/internship opportunities, and schools with co-ops most commonly had only 10–25% participation. A significant challenge that was cited to providing co-op experiences was the lack of local industry in which to place students. It was also noted that co-op experiences are not necessarily linked to the curriculum and/or student design projects. The most common approach to providing industrial context was to have projects and/or mentorship provided by industrial partners. Some programs have well-established systems for matching student teams with industry projects that in some cases involves a bidding process.

Potential barriers to developing these types of arrangements included conflicts over IP and mismatched expectations on both sides, *e.g.*, concerns about appropriate mentorship by the company or concerns about satisfactory work product from the team within the timeframe of the project.

The discussion focused on how to market the opportunity to industry and make it most attractive to them.

1. Opportunity to explore a new idea with low investment of resources.
2. Desire to contribute to education and talent pipeline development, including the chance to evaluate students as potential employees.
3. Alumni engagement.
4. It was noted that interdisciplinary teams are the norm in industry, so having interdisciplinary student teams might prove more attractive to industrial sponsors.

Finally, apart from direct involvement of external companies, familiarity with the concerns, constraints, and standards of industrial settings can be fostered by having industry professionals run the design program. It was also suggested to introduce design concepts early in the curriculum.

### Global Context

The general consensus of the participants was that study abroad achieves the goal of needs-finding in a global setting (72%) more effectively than lecturing (67%) and mentoring (58%). While participants acknowledged the value of a global immersion experience to the individual student, it was clearly a lower priority for most programs.^31^ This ranking is attributed to the perceived barriers to implementation that include high cost, lack of expertise, safety for students, and time. The existence and success of such programs usually depends on having one or more faculty devoted to doing it.

Without an immersion experience, exposure to the global context of a design project most commonly arises when a faculty member conducts research related to technologies that have a market in the developing world such that the context needs be considered in the design process.

It was also noted that “Global” need not refer exclusively to the developing world. It can also mean understanding local healthcare systems (rural or remote vs. urban) as well as differences in industry culture and practices. This provides students with experiences that help them to appreciate the opportunities and constraints for development and adoption of technologies. Some of the most challenging design constraints related to “global health” are “access to clinical care” and “availability of resources.” Both of these constraints are reproduced in some degree in rural healthcare facilities in impoverished regions of the USA. As such, this can serve as a viable substitute to teach many of the issues related to designing in a resource constrained environment.

The recent COVID pandemic events (occurring after the workshop) provide a concrete example of how a typical ’global health’ constraint such as ‘restricted travel’ and ‘availability of materials and supplies’ is evident locally. This potential local approach to global health issues was evident in our survey results, with one respondent stating that their design program seeks to “*Broaden perspective so that solutions meet the needs of underserved populations, or populations with different healthcare* / *economic pressures*”, and another respondent stating, “*We want all of our engineers to understand the global context and appreciate that medical devices and biomedical initiatives may function differently in different social/economic and political environments*.”

## WORKSHOP 2. EXPERIENTIAL LEARNING WITHIN COURSES

(Breakout Leaders: Joe Tranquillo, Beth Pruitt, and Glenn Gaudette)

This session explored ways to overcome the barriers to more widespread adoption of problem- and project-based learning (both often annotated as “PBL”) in BME classrooms.^1,14,19,21,23,46,49^ While similarities exist between these two methods, there are subtle but important differences.

In problem-based learning, the instructor provides a challenge that requires the students to learn the course material as they are solving the problem. Depending upon the complexity of the problem, it could be solved in a few minutes, one class, or over multiple classes. Like problem-based learning, project-based learning starts with a challenge, but unlike problem-based learning, project-based learning involves sustained inquiry and authenticity. Project-based learning also involves student choices, reflection, and revision.

In either case, the following characteristics exist:

(1) the assignments are often open-ended (and include real-world situations);
(2) the assignments are intentionally ill-defined;
(3) students are expected to identify, find, and use appropriate resources;
(4) students work in groups;
(5) learning is active, integrated, cumulative, and connected.

Prior to the workshop, 50 programs responded to a workshop survey about engagement and attitudes around PBL. For survey respondents, we defined PBL to include to include the elements 1–5 above, with an added component that:

(6) students must report the solutions.

With these 6 characteristics used to define PBL, programs were asked to report on the following:

- What percent of your courses use ALL 6 PBL elements in one experience?
  - Undergrad Core (required)
  - Undergrad (elective)
  - Grad Core
  - Mezzanine
- What percent of faculty teach PBL courses (with all 6 elements)?
  - Tenured/Tenure-track faculty?
  - Lecturers/Non-tenure track faculty
  - Industry/Guest
  - Others
- Where do the problems/projects originate? (select all that apply)
  - Faculty in department
  - Clinicians
  - Industry
  - Others
- What percent of these PBL-base courses result in a physical artifact vs. a paper-based design?
- At your institution, how important is incorporating PBL into courses? (Is it valued at your university and is it included in tenure and promotion?)
  - Scale: 1 to 10 where 0: Not valued, 5: Modestly valued, 10: Highly Valued

Within an overall curriculum, approximately 30% of required undergraduate and 24% of undergraduate elective courses use PBL. The use of PBL was much lower in graduate-level courses, with only 12% containing PBL. Of all courses using PBL, 65% require the creation of an artifact or prototype.

We note that active learning was not defined separately from PBL in the survey. Active learning could have included any of the aspects associated with PBL, as well as the use of other techniques such as quick-response, clickers, one-minute papers, think-pair-share, flipping, *etc.* As such, since PBL is a subset of active learning, we expect that a higher percentage of courses use some form of active learning than reported above.

From this survey, we learned that non-tenure track faculty (50%) were more likely than tenure track faculty (30%) to use some form of PBL. When asked to rank the perceived importance of PBL to their university, the average was 7 out of 10, (10 being most important). However, the range was enormous, spanning 1-10.

During the workshop, participants crowdsourced to name barriers to adopting PBL and then discussed ways to overcome these barriers. The key recommendations included:

1. starting small,
2. learning from other practitioners, and
3. explaining to students the rationale for active and team-based learning.

Below we summarize the topics discussed by participants in the workshop and the proposed solutions and rubrics for overcoming barriers to implementing BPL methods. Barriers and potential solutions clustered in the areas of a) how to build, manage and assess effective student teams, b) fear of the new and unfamiliar format, c) lack of resources, and d) cultural norms and value to faculty, students and the organization.

### Building and Managing Effective Student Teams

Participants discussed the challenges of how to build student teams thoughtfully and fairly, how to encourage effective, independent team management, and how to assess the learning outcomes of team-based assignments. Students often resist assigned teams, so it is worth reminding them that diverse teamwork experiences provide good preparation for industry jobs where managers will also dictate team composition and roles. Faculty may also find it helpful to emphasize that available time and commitment to work on a project is a stronger predictor of team success (grades) than existing friendships. A key recommendation of the discussion for team effectiveness was to invest the time up front in thoughtful team formation and transparent assessment rubrics, as described below.

Online team-maker and assessment tools can help faculty build a diversity of backgrounds and viewpoints in a team and ensure compatibility in student schedules and effort availability. Such tools often provide features for continuous online monitoring of team functioning through short surveys of team expectations at regular intervals.

Team sizes of 3 to 5 students promote team health, diversity and inclusion by avoiding problems of tokenism or isolating under-represented students without an ally. This size is large enough to resolve conflict (> 2) and small enough to avoid free-riders (< 6). Faculty can involve teams in their own governance by tasking students to create an asset map of team skills and to commit to a contract of team roles and how they will rotate through them. Team contracts provide a means for students to agree on what team members promise to do to compensate their teammates if they fail to deliver on assigned milestones.

For complex projects (e.g., interdisciplinary capstone design), faculty may wish to define key roles and rotation intervals to ensure students gain experience in new areas. Such contracts set expectations explicitly, provide a framework for formal team training on “soft-skills”, and a rubric for self-monitoring of team function and health. Some teams will experience problems and require intervention, but frequent monitoring will catch problems earlier. In larger courses, TAs can be strategically deployed to sit near, or work with, groups having functional problems and can provide the support or feedback to get them back on track.

Regular team evaluations also form a basis for performance assessments and eventual grading, and students should be aware of these uses. Differences between team (peer) and individual (self) assessments are not as common as faculty fear; they often provide critical opportunities to notice, and potentially rehabilitate, shallow learners. While setting up an assessment rubric and strategy for implementation requires an up-front investment of time and energy, regular assessment (with at least three milestones) is critical not only for team health but also for student perceptions of fairness in understanding how the instrument affects their grade. For example, the first assessment should come quickly after a small team milestone to serve as a no-risk practice with the instrument and as calibration to team expectations and scoring attitudes; the second assessment then provides meaningful feedback on how the team is sharing responsibility and is also an opportunity for mid-course counseling and corrective actions by those who fell behind expectations; the final assessment provides a summative evaluation.

### Instructor’s Fear of a New and Unfamiliar Format

Participants discussed the risks of letting students have autonomy and the possibility of failure. Up-front preparation by the teaching team, faculty and TAs, were recommended to scaffold the learning experience. For example, this preparation could include using frameworks and constraints to guide team direction and help them identify next critical steps, including defining roles, team checklists, milestones with deadlines, *etc*. The aim is for students to master these key skills and earn autonomy; this process can be aided by encouraging the use of moderated online platforms for students to ask questions and share knowledge. Participants also discussed fears around lack of faculty knowledge or experience. Where possible, faculty can gain confidence in PBL by pairing with faculty experienced in PBL through team-teaching or observation. University teaching centers often provide resources and training in how to get started with active learning and PBL. When departments wish to prioritize inserting more PBL into their curriculum, they can also incorporate an introduction to the benefits of PBL and a training session by an experienced faculty member during new faculty orientation or faculty retreats.

Research and training materials on PBL are readily available via the American Society for Engineering Education (ASEE) and its annual meeting, the annual ASEE National Effective Teaching Institute, the Frontiers in Education workshop, Kern Entrepreneurial Engineering Network (KEEN), and a number of similar training programs on PBL. These venues and their online literature and forums also provide resources for faculty to find ideas for PBL topics and successful rubrics for implementation. While many faculty members are comfortable drawing on their own real-life experiences or observing problems around them to devise problems, it may be beneficial to draw on problems from an industry network, from community resources or by engaging students in the needs-finding and problem ideation process. One recommendation emerging from the participants was for the creation of an online repository for formalized sharing of PBL materials and problems in the community.

### Lack of Resources

Participants discussed a lack of key resources as a barrier to implementing PBL in their classrooms. Foremost, faculty lack the resource of the time to “redo” their courses, to learn a new way of teaching, and to do the up-front work to create the PBL topics, frameworks and rubrics. Faculty who had adopted effective PBL strategies in their teaching generally find they and the students are more engaged and spend less time managing problems with student and team performance.

Participants who had used PBL successfully reflected that attending a workshop provided a dedicated time and support for the transition in their teaching philosophy. They strongly recommended that faculty should start small and not tear apart and redesign a course at once. Instead, they might start with one or two new in-class group exercises or modify some homework assignments to group problem-based learning activities that convene teams to work outside the classroom.

Participants discussed the perception that more TA support was needed to implement PBL. This is likely to be true during initial transitions; however, with time as faculty evolve their courses to include more PBL and active learning, they may have a pool of past students from their course who are familiar with PBL goals and practices, and it is expected that they may have an easier time recruiting engaged TAs and training them how to teach. Undergraduates can be recruited as a network of peer learning assistants, as has been done in programs like chemistry having large courses required for freshmen.^5^ In all cases, it was deemed essential to establish teaching team roles, clear expectations, regular teaching team meetings, and ways to manage and contact teaching team helpers.

Finally, a lack of flexible teaching spaces or prototyping facilities was viewed as an impediment to PBL. Participants reported overcoming this barrier by having students rearrange their seating or cluster in teams within fixed seating constraints and giving assignments for project work outside of the classroom. In the long term, faculty and departments can lobby for facilities and resources that support PBL such as room renovations and investments in flexible teaching spaces in new buildings. A key recommendation for effective use of resources for PBL was to start small and make use of what is available now.

### Cultural Norms and Value to Faculty, Students and the Organization

The last set of perceived barriers involved concerns with the acceptance and value of PBL to students, the department, and institution. Teaching evaluations by students often serve as the single snapshot of faculty teaching performance, and trying something new can be fraught with doubts about the perceptions of PBL effectiveness. Recommendations for dealing with negative student perceptions included (1) informing students up-front about motivations to make them partners in a new and innovative experience, and (2) engaging students in the benefits by citing PBL literature as evidence.

Not surprisingly, concerns of faculty resistance and peer perceptions of the value of PBL generated significant discussion related to the efforts required to change attitudes and institutional cultures. The same approaches used to influence students may serve to quiet any colleague with objections. For those committed to changing culture, participants also discussed strategies for supporting new faculty members or new courses by lobbying department chairs, deans and provosts so that they will recognize (and ultimately reward) teaching innovation through funding pilot projects, creating teaching innovation awards or by providing funding in startup packages earmarked for PBL teaching. A key recommendation from discussion of these challenges was that changing cultures is too high a bar and that merely convincing more resistant faculty not to object to PBL in your courses may be enough to get started.

### Survey Limitations and Further Approaches

This survey defined six elements of PBL for the respondents to consider when completing the survey. However, whether the respondents considered PBL as defined in the survey or based on their own understanding of PBL is unknown. Furthermore, the survey was not created specifically to assess the usage of PBL in Biomedical Engineering education, but rather to provide information to guide the workshop. Follow-up studies should better define the experiences and successes/challenges associated with each form of experiential learning. In depth assessment into the effectiveness of these pedagogical approaches is clearly a need that was expressed by members of this workshop and the reviewers of this paper.

For example, for project-based learning it is important to learn how the experience of creating a tangible product using a design, build and test experience motivates individuals and the group. Do the design experiences boost, or hinder, creativity? Does the timing of a project reinforce, or diminish, a desire to explore alternative approaches to solve a problem? Does the process induce a ‘just in time’ learning approach? If so, does that interfere, or reinforce the need to develop foundational knowledge?

For problem-based learning challenges that target engineering analyses, calculations, and documentation, many of the same questions are relevant. Additional questions might explore whether the lack of a working product end-goal improves the learning process by allowing students to focus on analyses, or diminish the experience by making the exercise more theoretical? Does the use of a documentation step improve students’ writing proficiency?

Finally, it will be worthwhile to explore whether either of these approaches work best for students of specific learning styles. If so, how can this method be most effectively implemented in groups with members of contrasting styles? Does the difficulty associated with incorporating students of different approaches make this an excellent teaching method for career development, or does it create a distraction from the deep learning of engineering practice?

## WORKSHOP 3. EXPERIENTIAL LEARNING IN CO-CURRICULAR LEARNING SETTINGS

(Breakout Leaders: Beth Winkelstein, Lee Makowski, and Jenny Amos)

This session aimed to explore experiences across the BME community related to co-curricular activities, with a focus on co-op, research, and global experiences. Survey results were collected from 60 respondents. Questions asked what types of experiences were offered in their program with follow-up questions requesting details associated with typical restrictions for participation, intended learning goals of the experiences, the percentage of students who participated, and the length of experience.

The workshop survey of programs showed that 25% offer co-op experiences, 40% offer research experiences, and 25% offer global experiences. Many used experiential learning as a way to expose students to ‘real-world’ problems and enhance professional skills such as communication, understanding of ethics and impact of designs, and to challenge the application of technical skills outside of the classroom.

After reviewing the results of the survey, an open discussion was held in the session asking participants for feedback on a summary of the data and to share any surprising insights. Participants were asked:

1. What are barriers to more participation (other than funding)?
2. How can credit be awarded for these activities?
3. Assessment—objectives and deliverables, and
4. How can we fund these activities?

The responses are summarized in Table 1.

A seldom recognized benefit of most co-curricular activities is the enhancement of the reputation of ’bioengineering’ or ’biomedical engineering’ as a discipline. Skepticism of employers about the benefits of a BME degree remains common. The larger co-op programs flourish by making significant outreach efforts to educate potential employers about the BME curriculum. Co-op also allows employers to assess the benefits of a BME curriculum directly through the hiring of a student under conditions that have little long-term risk. Experience suggests that once ’in the door’, BME students have proven very capable of convincing employers of the strengths and utility of their education. Global experiences and research in almost any format also enhance the attractiveness of students for potential employers. They are almost always more prone to hire students who can demonstrate some extended period of time working in a laboratory beyond those associated with courses.

Many co-curricular activities have a GPA cutoff. While this is often used to aid in identification of those students who may perform best in a particular activity, it was pointed out that it was arbitrary and might often be used to exclude those students who could benefit the most. Furthermore, there is evidence that the value is higher to first-generation, low-income, under-represented students who may not have high GPAs but excel by other metrics. The sense of the discussion was that institutions should reconsider GPA as a criterion for participation or use it as one of only a number of criteria. Research indicates that experiential engineering education programs can provide an excellent environment in which to observe and measure and facilitate students developing and demonstrating engineering competencies.^27^

Co-op and internship opportunities can significantly enrich engineering education and enhance students’ skill development.^27^ However, in order to optimize the impact of the co-op experience, it is critically important to prepare the student for the experience and provide support and guidance. In general, a work placement will be of greater value if it gives students a chance to put into practice what they have learned in the classroom and if the placement is longer than is typical for a summer job or internship.^3^

When faculty participate in offering co-curricular activities there often arises a question as to training required for their participation and compensation for their time and efforts. It appears that faculty compensation is frequently not taken into account in the design and offering of co-curricular activities, especially when mentoring student research. Mentoring of student co-curricular activities often occurs without recognition in the form of credit or pay, particularly if there is a graded component. For global experiences, a faculty member is often traveling and working during breaks (winter/summer) and may not receive credit towards teaching to compensate for their efforts.

Discussion of cross-disciplinary experiences raised the question of how to support students doing research in other fields or during cultural immersion. These experiences are common and considered highly beneficial, but such experiences may be perceived as peripheral to their engineering education. Discussion emphasized the institutional commitment/support needed to allow the faculty and students participating the flexibility needed for a global experience. More pressing issues that were mentioned but not discussed extensively were identification, evaluation and placement of hosts and quantification of the value of the experience.

Action items that were identified for follow up included:

- Faculty culture does not always support participation in these experiences. Faculty may not know about, or recommend, these types of experiences to students. If they are promoted and encouraged, they are often not brought back into the curriculum in any way. Examples of practices that embrace co-curricular experiences are giving credit (beyond non-degree hours) and portfolios that include reflection.
- There is a lack of assessment in co-curricular activities. Examples of needs are templates for courses that can award credit and assessments that are related to engineering knowledge and skills, not just program satisfaction.
- Faculty compensation and institutional support for flexibility in curriculum to allow for these experiences is needed. Programs that can offer co-curricular activities every semester will likely have higher rates of participation; further, programs that offer co-curricular activities over the summer will likely have the highest participation. Future collation of data regarding the effectiveness of co-curricular activities would provide much-needed rationale for additional course offerings.

## WORKSHOP 4. TEAM-BASED LEARNING

(Breakout Leaders: Ann Saterbak, Donald Gaver, and Joe LeDoux)

The participants in this session discussed the value and complications associated with team-based learning. Survey data indicate that the preponderance of BME programs rely on team-based learning, and many challenges exist in teaching this process and evaluating student outcomes. In this session, four affinity working groups discussed key issues and solutions related to teaching, motivating, moderating, and evaluating team-based learning in BME. Papers associated with this topic are provided as references in the following areas:

- purposes of teams,^50,2,40^
- teaching teaming,^54,59,51,53,6,22,52,55^
- team challenges,^41,39,60,16,12,17,15^ and
- team composition.^32,13,62,56,29^

Pre-conference workshop survey results reinforce the importance of team-based learning in BME. These data show that the majority of programs require teamwork during at least three years of the undergraduate curriculum, with at least six classes (and frequently 10 or more classes) requiring teamwork. Key challenges include scheduling (25%), conflict management (20%), personality conflict (20%), social loafing (18%), project management (12%), and role assignment (6%).

Session participants were subdivided into affinity groups with 5–7 participants in each group to discuss four major issues associated with team-based learning: teaching and learning teamwork, social loafing, team conflict and team evaluation.

### Teaching and Learning Teamwork

The participants in this affinity working group discussed best practices for scaffolding knowledge about teamwork in our students. The discussion focused on having students learn about the concepts and issues related to teamwork and the intrinsic value and risks associated with the teamwork process. The participants agreed that issues to teach are motivation, responsibility, team roles, conflict resolution and intra-team communication. It was recognized that teamwork experiences are a critical component of undergraduate education, with students becoming aware of how to work in diverse teams.

Workshop participants concluded that the teamwork concepts are best taught through in-class simulations with well-structured experiences. In these experiences, the teamwork rubric can be introduced, and students should experiment with alternative team roles, team management, accountability, leadership, and communication. Post-simulation reflection and evaluation processes were seen as critically important aspects of the learning process. Students may benefit from resources for learning effective teamwork (see bibliography) which could be discussed in class.

### Social Loafing

Here “Social Loafing” describes the tendency of individuals to put forth less effort when they are part of a group. This can occur if members of the group pool their effort to achieve a common goal, and members contribute less than they would if they were individually responsible. This affinity group identified causes, enabling behaviors, and successful intervention methods for social loafing.

Causes and enabling factors include an overemphasis on the final project rather than the process and short-term goals. Students’ lack of interest may result from a misunderstanding of the added value for the team process and accountability issues (e.g., ‘friends don’t rat out friends’). In addition, poor team development can lead to communication errors, non-inclusive groups, and ineffective team dynamics.

It was agreed that instructors may partially inoculate students from social loafing by preemptively discussing team dynamic models with an open eye to a discussion of teamwork pitfalls. Students could then decide upon their operating principles as a course deliverable—this would include the students’ self-determination of team assignments, performance norms, and meeting times and locations. Clear assessment was seen as a must, with a key aspect being the use of a logbook that reports individual contributions that is reviewed and agreed upon by all team members. To reinforce the need for student participation, the instructor(s) should meet with individual students as well as teams, and grades should reinforce accountability by including individual and team components. Some faculty preferred to have students define their own teams, whereas others used published tools, methods or heuristics. Having students define their own teams can provide them with the experience of learning to form teams with the deep level diversity needed to successfully accomplish its objectives. However, teams formed by students, if allowed to proceed without guidance and support from the instructor, could result in teams that are more cohesive but less diverse and therefore prone to group-think. It is generally regarded as a best practice that instructor(s) should define the group makeup, possibly using CATME software (https://www.catme.org) to aid in the selection process. Best practice dictates that students who are under-represented in a field (*e.g.*, women, students of color) should not be isolated (i.e., one African-American woman with four Caucasian men).

### Team Conflict

The working groups identified likely areas of conflict due to macro-conditions such as high-stress environment, misunderstood objectives and scheduling conflicts. At the student level, conflicts exist because of differences in commitment, expertise/skills imbalances, personality conflicts, communication breakdowns and leadership difficulties. Instructors should participate in conflict resolution through clear descriptions of course expectations and conflict management.

It was agreed that it is important for students and instructors alike to learn to accept conflict as part of the nature of teamwork. To best negotiate this process, it is important for instructors and students to preemptively discuss potential conflicts and define the conflict resolution process—this process should preserve group autonomy and emphasize early intervention. These skills can be taught through team-building exercises that would lead students to define their roles and leadership structure. This process could aid in students’ ability to recognize and value workstyle differences. Instructors should be responsible for early and frequent communication with checkpoints that should focus on results with clear expectations.

### Team Evaluation

In this component, the affinity group addressed the many forms of team evaluation and how these can be used to incentivize high performance. As with the other affinity groups, the participants believe that teamwork should be emphasized in the course’s evaluation rubric. The participants reinforced the use of team-derived contracts, ideally with defined roles for students.

It was agreed that thoughtful peer review is an essential component of the evaluation; however, this can be problematic if students try to ‘game the system.’ Assessments should include formative and summative individual self-evaluation, peer-to-peer assessment, and group assessment. To maintain high quality and student engagement, feedback should be timely and consistent and impact student final grades. CATME is widely used for this purpose.

In summary, team-based learning is an essential and frequently used component of the teaching/learning process in BME. Successful teaching of team-based learning processes can reinforce the importance and value of learning styles, diversity, delegation and responsibility, and project completion. Teaching and managing team-based learning would benefit from instructional materials to teach teamwork processes through role-playing exercises. Instructors are responsible for early and frequent communication with checkpoints that should focus on results with clear expectations. Student performance will benefit from the implementation of a carefully designed assessment rubric that includes formative and summative peer-to-peer evaluation, individual self-evaluation, and team assessment.

## WORKSHOP 5. TEACHING TO REACH A DIVERSE CLASSROOM

(Breakout Leaders: Brian Helmke, Michele Grimm, and Paul Benkeser)

The general goal of this workshop was to identify and share common practices and resources in BME programs that support a diverse population of BME students. Although the undergraduate student population in BME contains a higher proportion of women and members of underrepresented groups than in other engineering disciplines, these proportions are lower than in society as a whole.^9^ Strategies for identifying barriers to participation and for promoting inclusion of underrepresented groups in BME have been proposed.^9,10^ Similar evidence-based practices designed to support students’ variability have been proposed as strategies to improve learning in the classroom.^61^ However, assessment of the level of implementation of these strategies in BME departments has not yet been completed.

An emergent theme in the pre-workshop survey data was the need to include intentional strategies supporting an inclusive classroom environment, especially when using collaborative learning or group work. As a result, the workshop was designed with the following learning objectives for participants:

1. to identify and begin to address inclusiveness challenges in their courses,
2. to reflect on how group formation methods may be inclusive or isolating,
3. to recognize inclusive practices in the classroom, and
4. to leverage existing resources that support best practices for inclusive teaching.

The workshop was designed as a collaborative learning activity for participants. Participants self-formed discussion groups of 3–4 members. Participant responses to prompts during the workshop were collected using QuestionPress (www.questionpress.com). Since one common strategy for promoting diversity identified in the pre-workshop survey was to assign group projects, participants first reflected on their own experiences with group projects as students. The discussion included types of learner variability to consider during group projects, benefits of group work for learning, and Universal Design for Learning as a framework for inclusive teaching.^7,34^

Participants put these concepts into practice through a series of three case studies presenting scenarios from a hypothetical class as follows.

### Case Study 1

Prof. Christian Amaro is teaching a 2000-level introduction to biomedical engineering course. The course has approximately 100 students and is held in an auditorium. Prof. Amaro had students complete a survey at the beginning of the semester to help get to know the students better. The class has a handful of first-generation and transfer students, a couple of student athletes, a few more international students, and a sizeable fraction of students who entered university with AP credits in physics, biology, chemistry, calculus and computer science. There are also five students with accommodations that include extended time on assignments, extended absences, and a notetaker for class work.

#### Discussion Questions

What challenges will Prof. Amaro want to overcome to make sure all students have equitable access to learning opportunities? How might Prof. Amaro overcome those challenges?

#### Responses

Participants identified challenges to overcome, including class size, variability in student background and experiences, and students’ requests for accommodations. Discussion of strategies to address these challenges centered around two main themes. First, the instructor should promote feelings of belonging by creating a positive social and emotional climate in the classroom and by helping students manage feelings of stress and threat. The instructor could create a supportive environment by conveying that variable backgrounds and experiences are valued, by removing cues that trigger worries about stereotypes, and by emphasizing the learning purposes of activities and assignments. The instructor might foster peer discussions so that students recognize they are not alone in their feelings of stress. Moreover, the instructor should express confidence in students’ abilities to achieve high standards in the class. The goal is to promote a growth mindset, increasing a students’ confidence in their abilities to learn and to succeed in the class.

The second discussion theme focused on improving accessibility of both course materials and the physical classroom space. The format of course materials should respond to a broad range of learning preferences and should be accessible to all students with a range of abilities. Providing materials in written, visual, and audio formats allows students to interact with materials according to their preferences. Enabling subtitles and supporting screen readers are two ways to improve accessibility for all students. The physical classroom space should be compliant with provisions of the Americans with Disabilities Act. Helpful features include space for instructors and students to move around the room (to interact comfortably or for self-care without disruption), friendly sight lines, and tables with power and Wi-Fi to work at.

### Case Study 2

This semester, Prof. Amaro has used group work both in and out of class. Throughout the semester, Prof. Amaro has set up clear expectations for in-class group work, which typically entails small group problem-solving assignments. Students know they should engage with their peers and write their own answers to turn in. Prof. Amaro has a group activity planned for the final lecture of the semester that is similar to what students have done throughout the course.

#### Discussion Question

How is Prof. Amaro’s plan consistent with inclusive practices?

#### Responses

Participants noted that clear expectations, practice activities that increase familiarity, and team-based learning are features that increase feelings of belonging and self-efficacy. Students are therefore likely to experience an increase in motivation to participate. These group work activities also serve to scaffold the upcoming final lecture of the semester, increasing students’ chances of success.

### Case Study 3

After 15 minutes of review material, Prof. Amaro moves into the group activity. For this activity, students have a design problem that they have to discuss and then answer individually. The students access the worksheet online while the professor reads the problem on the board to the students and the TAs hand out paper copies to those who want them. Prof. Amaro asks students to form groups of 2–3 to discuss the problems.

#### Discussion Questions

What aspects of this activity increase inclusiveness for all students? What concerns about inclusiveness do you have as the activity proceeds?

#### Responses

Participants identified classroom practices that increase or decrease inclusiveness for all students. Inclusiveness was increased by starting with a warmup review activity and by providing the activity worksheet in multiple ways (verbally, online, on paper). Other suggestions for increasing inclusiveness included providing avenues for all voices to be heard by giving time for groups to establish a process for their discussions, by assigning and rotating roles during group activities, and by providing or co-creating discussion guidelines that all allow all group members to speak. Inclusiveness may or may not be affected by the method of group formation and the group size. For example, self-assembling groups randomly may present barriers to students with less sense of belonging; on the other hand, frequent self-assembling of groups may normalize the uncertainty and provide an avenue to interact with more classmates.

Finally, discussions by workshop participants reviewed recommendations and resources for designing inclusion into courses available in an “Inclusion by Design” worksheet (http://bit.ly/inclusionbydesign).^35^ This topic overlaps the discussion in Workshop 6: Innovative platforms and pedagogy. References ^30,43,64,65,66,67,68,69,70,71,72^ provide links to resources at a number of institutions with BME departments. Further references provide information on how to support best practices in

- effective team-based instruction^42,48,57^
- collaborative learning,^4,36,18^
- inclusiveness,^7,34^
- team formation,^24,28,44^ and
- technology-based audience-response tools.^25,33,45,47,58,63^

A post-workshop survey indicated that a majority of BME programs leverage institutional resources to support students with diverse backgrounds, experiences, and learning needs. However, the implementation of strategies to support student variability in the classroom varies widely among programs and individual instructors. Most workshop participants felt that the workshop helped them learn more about attending to student learning needs and supporting group work. It was suggested that the Biomedical Engineering Society (BMES) should collaborate with the American Society of Engineering Education (ASEE) to faculty development in this area.

## WORKSHOP 6. INNOVATIVE PLATFORMS AND PEDAGOGY

(Breakout Leaders: LeAnn Dourte Segan, Bryan Pfister, David Meaney, and Treena Arinzeh)

The goal of the workshop was to determine best practices in innovative platforms and pedagogy and to better understand their usage in fostering life-long learning and teaching to a diverse audience. Chairpersons were asked survey questions prior to attending the workshop:

1. Have the faculty in your department been encouraged to attend professional development workshops on new teaching platforms?
2. Does your BE/BME program use innovative techniques in teaching to help meet the needs of culturally diverse students?

In addition, they were asked to provide examples of workshops or techniques relevant to each question.

The workshop survey results revealed that the majority of chairpersons do encourage faculty to participate in workshops (Yes: 79%, No: 21%). Common examples were NETI (National Effective Teaching Institute), KEEN (Kern Entrepreneurial Engineering Network), and national- and university-supported workshops on hybrid, active and flipped courses. Therefore, we sought to gain a better understanding of the implementation of these techniques in the classroom.

Fewer than half of the chairpersons responded that their programs use innovative techniques to help meet the needs of culturally diverse students (Yes: 43%, No: 57%). For those who did respond “yes”, the term “culturally diverse students” appeared to have varied definitions depending upon the institution, and it was unclear how the teaching platforms supported these students.

Based on these survey data and analyses, two main questions were asked of workshop attendees:

1. How can we support and encourage faculty to implement evidence-based pedagogy and use innovative platforms?
2. How do we use innovative platforms and pedagogy to engage a diverse audience?

Many instructors feel undereducated and are concerned about the time needed for successful implementation. A common theme was that departments should make teaching a priority and develop a culture of teaching (*e.g.*, discuss at faculty meetings, identify good teachers and internal experts, assign teaching mentors). However, attendees questioned the roles of instructional faculty and research faculty when it comes to educational innovation. It was suggested that instructional faculty could be used to drive innovation changes. Developing ways to incentivize faculty to learn and incorporate evidence-based teaching methods are needed. Incentives could include release time, teaching/course grants, and teaching the same course multiple times. At the university level, classroom infrastructure would need to be improved to implement many innovative approaches.

Diversity in the student population was agreed to have a broad definition (*e.g.*, race, ethnicity, learning abilities, mental health challenges, first generation, socioeconomic status) and the participants agreed that there is insufficient faculty training/education to support diversity. To foster life-long learning for diverse students, pedagogical approaches such as PBL and active learning are excellent ways to introduce students to real-world problem solving that involves the process of acquiring and applying new knowledge. However, implementation is an issue. Some concerns included concerns of covering the same amount of course material as in a lecture-based format (depth *vs*. breadth) and a need to identify best methods to give feedback and assess students beyond traditional “textbook problems.” Best practices included using non-graded homework with in-class discussion and considering online assessment programs. The audience also agreed it takes additional resources for implementing these teaching techniques. Consideration should be given to collecting and promoting evidence-based teaching methods that do not require significant resources. Suggestions were using undergraduate peer learning assistants, creating “packaged teaching tools” that can be used department-wide, and providing techniques in a format that can be easily implemented into lecture-based courses.

Overall, *faculty training and implementation* remain the major obstacles to the use of evidence-based pedagogy and innovative platforms. These barriers exist due to faculty and institutional priorities as well as costs/resources. While many teaching innovations have been proposed, faculty still find it difficult to deliver course content to a diverse student body with time-intensive methodologies. Overcoming these challenges would better enable instructors to foster life-long learning and support a diverse audience. It is recommended that a cross-institution study be conducted to determine the number of faculty participating in these workshops and the fraction who subsequently implement the techniques in the classroom.

## CONCLUSIONS

Participants of the Learning Environments converged on a few central themes, indicating the importance of (1) fostering inclusive teamwork for our students, (2) supporting authentic curricular and co-curricular project experiences, and (3) incentivizing and supporting our faculty to adopt best practices for inclusive and active learning in their classrooms.

Based on the pre-summit surveys, BME programs appear to broadly implement team-based learning, and two urgent needs emerged:

1. coaching faculty in this format, and
2. more sophisticated assessments of individual student performance.

Survey results indicated that tenure track faculty were far less likely to use active-learning approaches than their non-tenure-track colleagues, and it would be wise to determine whether institutional cultures can be modified to improve the adoption of active-learning.

Faculty need ready access to effective tools to help them form diverse teams of students, avoid social loafing, and facilitate peer-feedback. Second, while the majority of participants in the sessions recognize the value for authentic, real-world project experiences for their students and the need to engage their students in active format within the classroom, many faculty members have not been trained in using best practices to optimize the value of projects. Faculty should be encouraged to attend the many excellent sessions at ASEE and BMES annual conferences where innovations and best practices in project advising are discussed. Finally, a consensus emerged that faculty require specific and frequent professional development opportunities and dedicated time in their schedules to adopt these best practices and they should search out workshops provided by their universities and others such as the Center for Project-Based Learning (https://wp.wpi.edu/projectbasedlearning/).

Participants concluded that a scholarly approach should be used to assess the value of new methods for developing and implementing team projects. Cross-institution studies could aid in cataloging the barriers and incentives for implementation of best practices, and surveys would allow us to determine the number of faculty who participate in teaching workshops and subsequently implement the techniques in the classroom. At each institution, it is advisable for administration to provide their faculty with an understanding of the:

1. incentive structures related to adoption of active and inclusive teaching, including formalized training, teaching buy-out and funds for workshop registration, and
2. how innovative and inclusive practices can be included in annual reports and evaluation metrics for tenure and promotion.

It may also be useful to determine the willingness of administration to redesign course student evaluations so that the impact of innovative practices can be evaluated.

In summary, due to the inherently interdisciplinary and dynamic character of BME, project experiences and working in diverse teams are especially important for the success of our students. While educational best practices are not unique to our field, we have the opportunity and obligation to implement inclusive team-based projects throughout our curricula to ensure that all BME students develop the skills and knowledge to identify and solve the complex problems of tomorrow.

## Figures and Tables

**TABLE 1.** Unique challenges for different types of co-curricular experience.

Co-op experience	Research experience	Global experience
Curricular integration and how to support students who are out of sync with curriculum International students are hard to place and can’t be paid due to visa issues	Course credit and structure—many are awarding some form of credit but without consistent assessment of work and lack of consistency in deliverables and expectations for work	Costs—international travel is hard to fund, NSF and NIH very limited Length—how long does a student need to go to benefit? Is a one-week experience sufficient? Do students need a semester or year?

## References

[1] Allen DE, Donham RS, Bernhardt SA. Problem-based learning. New Dir Teach Learn. 2011;2011(128):21–9.

[2] Artz GM, Jacobs K, Boessen CR. The whole is greater than the sum: an empirical analysis of the effect of team based learning on student achievement. NACTA J. 2016;60:405–11.

[3] Baber T, Fortenberry N. The academic value of cooperative education: a literature review. In: Proceedings for the American Society for Engineering Education annual conference, Pittsburgh, PA, USA, June 22, vol. 25; 2008.

[4] Barkley EF, Cross KP, Major CH. Collaborative learning techniques: a handbook for college faculty. 2nd ed. San-Francisco: Jossey-Bass; 2014.

[5] Berkeley College of Chemistry, Teacher-Scholars Program (COCTSP); 2021. https://chemistry.berkeley.edu/ugrad/current-students/teacher-scholars.

[6] Blair GM. Laying the foundations for effective teamwork. Eng Sci Educ J; 2009.

[7] Burgstahler SE, editor. Universal design in higher education: from principles to practice. 2nd ed. Cambridge, MA: Harvard Education Press; 2015.

[8] Chatterjee D, Ford JK, Rojewski J, Watts SW. Exploring the impact of formal internships on biomedical graduate and postgraduate careers: an interview study. CBE Life Sci Educ. 2019;18(2):ar20. 10.1187/cbe.18-09-0199”. 31074697 PMC6755228

[9] Chesler NC. A how-to guide for promoting diversity and inclusion in biomedical engineering. Ann Biomed Eng. 2019;47:1167. 10.1007/s10439-019-02223-2. 30746597 PMC6456400

[10] Chesler NC, Barabino G, Bhatia SN, The pipeline still leaks and more than you think: a status report on gender diversity in biomedical engineering. Ann Biomed Eng. 2010;38:1928. 10.1007/s10439-010-9958-9. 20162356

[11] Cordray DS, Harris TR, Klein S. A research synthesis of the effectiveness, replicability, and generality of the VaNTH challenge-based instructional modules in bioengineering. J Eng Educ. 2009;98:335–48. 10.1002/j.2168-9830.2009.tb01031.x.

[12] Dayan M, Di Benedetto A. Procedural and interactional justice perceptions and teamwork quality. J Bus Ind Mark. 2008;23:566–76.

[13] van Dijk H, Meyer B, van Engen M, Loyd DL. Microdynamicsin diverse teams: a review and integration of the diversity and stereotyping literatures. Acad Manag Ann. 2017. 10.5465/annals.2014.0046.

[14] De Graaf E, Kolmos A. Characteristics of problem-based learning. Int J Eng Educ. 2003;19(5):657–62.

[15] Hackman JR. Leading teams: setting the stage for great performances. Boston: Harvard Business School; 2002.

[16] Haller CR, Gallagher VJ, Weldon TL, Felder RM. Dynamics of peer education in cooperative learning workgroups. J Eng Educ. 2013. 10.1002/j.2168-9830.2000.tb00527.x.

[17] Hodges LC. Contemporary issues in group learning in undergraduate science classrooms: a perspective from student engagement. CBE Life Sci Educ. 2018;17(2):es3. 29749840 10.1187/cbe.17-11-0239 PMC5998321

[18] Khourey-Bowers C Structured academic controversy; 2018. https://serc.carleton.edu/sp/library/sac/index.html. Accessed 30 Jan 2019.

[19] Kokotsaki D, Menzies V, Wiggins A. Project-based learning: a review of the literature. Improv Sch. 2016;19(3):267–77.

[20] Kotche M, Felder AE, Wilkens K, Perspectives on bioengineering clinical immersion: history, innovation, and impact. Ann Biomed Eng. 2020;48:2301–9. 10.1007/s10439-020-02508-x. 32314300 PMC7452935

[21] Krajcik JS, Phyllis CB. Project-based learning. In: Cambridge handbook of the learning sciences, Chapter 19; 2006.

[22] Laeser M, Moskal BM, Knecht R, Lasich D. Engineering design: examining the impact of gender and the team’s gender composition. J Eng Educ. 2003. 10.1002/j.2168-9830.2003.tb00737.x.

[23] Larmer J, Mergendoller JR. Seven essentials for project-based learning. Educ Leadersh. 2010;68(1):34–7.

[24] Layton RA, Loughry ML, Ohland MW, Ricco GD. Design and validation of a web-based system for assigning members to teams using instructor-specified criteria. Adv Eng Educ. 2010;2(1):1–28.

[25] Learning Catalytics. Interactive audience response using any web-enabled device. https://learningcatalytics.com.

[26] Linsenmeier RA. The *de facto* core curriculum in BME and BioE. 4th BME Education Summit, Cleveland, OH, 29–31 May; 2019. https://www.bmes.org/files/Robert%20Leisenmeier%20Presentation.pdf.

[27] Liu Q, Kovalchuk S, Rottmann C, Reeve D. Engineering co-op and internship experiences and outcomes: the roles of workplaces, academic institutions and students. Paper presented at the annual conference of the Canadian Engineering Education Association, Vancouver, British Columbia, Canada; 2018. https://hdl.handle.net/1807/94141.

[28] Loughry ML, Ohland MW, Moore DD. Development of a theory-based assessment of team member effectiveness. Educ Psychol Measur. 2007;67(3):505–24.

[29] Lvina E, Johns G, Vandenberghe C. Team political skill composition as a determinant of team cohesiveness and performance. J Manage. 2018. 10.1177/0149206315598371.

[30] Marquette Univ https://www.marquette.edu/center-for-teaching-and-learning/inclusive-teaching-resources.php.

[31] McCullough M, Msafiri N, Richardson WJ, Harman M, DesJardins JD, Dean D. Diversifying bioengineering design education with an international partnership. J Biomech Eng. 2019;141(12):1245031–8. 10.1115/1.4045112. PMC7104770 31596921

[32] Mello AL, Rentsch JR. Cognitive diversity in teams: a multidisciplinary review. Small Gr Res. 2015;46:623–58.

[33] Mentimeter. Interactive presentation web tool. https://www.mentimeter.com.

[34] Moore SL. Universal design for learning: online tutorial. (2007). http://www.hyperformer.com/UDL_tutorial/. Accessed 30 Jan 2019.

[35] Moore CS, Brantmeir E, Brocheild A. Faculty focus. (2017). https://www.facultyfocus.com/articles/course-design-ideas/inclusion-by-design-tool-helps-faculty-examine-teaching-practices/.

[36] National Institute for Science Education. Doing CL; 1997. http://archive.wceruw.org/cl1/CL/doingcl/DCL1.asp. Accessed 30 Jan 2019.

[37] Newstetter WC. Fostering integrative problem solving in biomedical engineering: the PBL approach. Ann Biomed Eng. 2006;34:217–25. 16496085 10.1007/s10439-005-9034-z

[38] Newstetter WC. Cognition and learning at the crossroads of the biosciences, engineering, and medicine. In: 4th BME education summit, Cleveland, OH, May 29–31; 2019. https://www.bmes.org/files/BME%20Educational%20Summit.pdf..

[39] Nokes-Malach TJ, Meade ML, Morrow DG. The effect of expertise on collaborative problem solving. Thinkin and Reason. 2012;18(1):32–58.

[40] Nokes-Malach TJ, Richey JE, Gadgil S. When is it better to learn together? Insights from research on collaborative learning. Educ Psychol Rev. 2015;27:645–56.

[41] Nokes-Malach TJ, Richey JE, Gadgil S. When is it better to learn together? Insights from research on collaborative learning. Educ Psycho Rev. 2015. 10.1007/s10648-015-9312-8.

[42] Oakley B, Felder RM, Brent R, Elhajj I. Turning student groups into effective teams. J Stud-Center Learn. 2004;2(1):9–34

[43] Ohio State Univ. https://ucat.osu.edu/inclusive-teaching/what-is-inclusive-teaching/.

[44] Ohland MW, Loughry ML, Woehr DJ, Finelli CJ, Bullard LG, Felder RM, Layton RA, Pomeranz HR, Schmucker DG. The comprehensive assessment of team member effectiveness: development of a behaviorally anchored rating scale for self and peer evaluation. Acad Manag Learn Educ. 2012;11(4):609–30.

[45] Poll Everywhere. Interactive audience participation presentation tool. https://www.polleverywhere.com.

[46] Prince M, Felder R. The many faces of inductive teaching and learning. J Coll Sci Teach. 2007;36(5):14.

[47] QuestionPress. Interactive audience response using any web-enabled device. http://www.questionpress.com.

[48] Reis R. Tips and strategies for effective teamwork; n.d. https://tomprof.stanford.edu/posting/1045. Accessed 30 Jan 2019.

[49] Savin-Baden M, Major CH. Foundations of problem-based learning. UK: McGraw-hill Education; 2004.

[50] Schaefer GC, Eckert C, editors. Design education today. Cham: Springer International Publishing; 2019.

[51] Schultz T Practical problems in organizing student into groups. In: Proceedings of IEEE Computer Society symposium frontiers in education, Tempe, AZ; 1998 p. 242–5. 10.1109/FIE.1998.736841.

[52] Seat E, Lord SM. Enabling effective engineering teams: a program for teaching interaction skills. J Eng Educ. 2013. 10.1002/j.2168-9830.1999.tb00463.x.

[53] Seat E, Parsons JR, Poppen WA. Enabling engineering performance skills: a program to teach communication, leadership, and teamwork. J Eng Educ. 2013. 10.1002/j.2168-9830.2001.tb00561.x.

[54] Sharp JE. Teaching teamwork communication with Kolb learning style theory. In: 31st Annual frontiers in education conference. Impact on engineering and science education. Conference proceedings (Cat. No.01CH37193), Reno, NV; 2001, p. F2C–1. 10.1109/FIE.2001.963699.

[55] Sharp J Learning styles and technical communication: improving communication and teamwork skills. In: Proceedings of IEEE computer society symposium frontiers in education, Tempe, AZ; 1998, p. 512–7. 10.1109/FIE.1998.736906.

[56] Shemla M, Meyer B, Greer L, Jehn KA. A review of perceived diversity in teams: does how members perceive their team’s composition affect team processes and outcomes? J Organ Behav. 2016. 10.1002/job.1957.

[57] Shimazoe J, Aldrich H. Group work can be gratifying: understanding & overcoming resistance to cooperative learning. Coll Teach. 2010;58(2):52–7.

[58] Slido. Q&A and polling, includes voting on user-entered items. http://www.slido.com.

[59] Smith KA. Strategies for developing engineering student’s teamwork and project management skills. In: Proceedings of the 2001 American Society for Engineering education annual conference & exposition; 2001.

[60] Smith KA. Cooperative learning: effective teamwork for engineering classrooms. In: Proceedings frontiers in education 1995 25th annual conference. Engineering education for the 21st century, Atlanta, GA; 1995, vol. 1, p. 2b5.13–2b5.18. 10.1109/FIE.1995.483059.

[61] Tanner KD. Structure matters: twenty-one teaching strategies to promote student engagement and cultivate classroom equity. CBE Life Sci Educ. 2013;12:322–31. 10.1187/cbe.13-06-0115. 24006379 PMC3762997

[62] Tekleab AG, Karaca A, Quigley NR, Tsang EWK. Re-examining the functional diversity-performance relationship: the roles of behavioral integration, team cohesion, and team learning. J Bus Res. 2016;69:3500–7.

[63] Top Hat. Integrated suite of web tools for content delivery, interaction, and assessment. https://tophat.com.

[64] Univ. of Arizona. https://diversity.arizona.edu/creating-inclusive-classrooms.

[65] Univ. of California, Los Angeles. https://ceils.ucla.edu/resources/teaching-guides/inclusive-teaching-for-diverse-classrooms/#toggle-id-7.

[66] Univ. of Florida. http://teach.ufl.edu/resource-library/inclusivity-in-the-classroom/.

[67] Univ. of Michigan. http://www.crlt.umich.edu/multicultural-teaching/inclusive-teaching-strategies.

[68] Univ. of Pennsylvania. https://www.ctl.upenn.edu/inclusive-teaching.

[69] Univ. of Texas at Austin. https://facultyinnovate.utexas.edu/inclusive.

[70] Univ. of Washington. http://www.washington.edu/teaching/teaching-resources/inclusive-teaching-at-uw/inclusive-teaching-strategies/.

[71] Vanderbilt Univ https://cft.vanderbilt.edu/guides-sub-pages/increasing-inclusivity-in-the-classroom/.

[72] Washington Univ. in St. Louis. https://teachingcenter.wustl.edu/resources/inclusive-teaching-learning/strategies-for-inclusive-teaching/.

[73] White JA, Gaver DP, Butera RJ, Core competencies for undergraduates in bioengineering and biomedical engineering: findings, consequences, and recommendations. Ann Biomed Eng. 2020;48:905–12. 10.1007/s10439-020-02468-2. 32026231 PMC13082399

